# Supplementary material for: Application of the Key Characteristics of Carcinogens to Per and Polyfluoroalkyl Substances
Source: Int J Environ Res Public Health. 2020 Mar 4;17(5):1668. doi: 10.3390/ijerph17051668 (PMC7084585; doi:10.3390/ijerph17051668)
Supplement: Supplementary file 1 [file ijerph-17-01668-s001.pdf]

Table S1. List of abbreviations for reviewed PFAS and data availability for key characteristics.

Abbreviation	Chemical Name	PFAS Type	Chemical Formula	CAS Number	Key characteristics of carcinogens (1-10) assessed for individual PFAS in different types of studies		
	**PFOA**				**Epidemiological**	**Animal Bioassay**	**In vitro**
PFOA	Perfluorooctanoic acid	Perfluoroalkyl carboxylic acid	$C_8HF_{15}O_2$	335-67-1	1, 4, 5, 6, 7, 8, 9	2, 5, 6, 7, 8, 10	2, 4, 5, 6, 7, 8, 10
	**Long Chain PFAS**				**Epidemiological**	**Animal Bioassay**	**In vitro**
PFOS	Perfluorooctane sulfonic acid	Perfluoroalkane sulfonic acid	$C_8HF_{17}SO_3$	1763-23-1	1, 4, 5, 6, 7, 8, 9	2, 4, 5, 7, 9	2, 4, 5, 6, 7, 8, 10
PFHxS	Perfluorohexane sulfonic acid	Perfluoroalkane sulfonic acid	$C_6HF_{13}SO_3$	355-46-4	1, 4, 5, 7, 8, 9	2, 8, 10	2, 5, 6, 8
PFNA	Perfluorononanoic acid	Perfluoroalkyl carboxylic acid	$C_9HF_{17}O_2$	375-95-1	4, 5, 7, 8	2, 5, 9, 10	2, 5, 8
PFDA	Perfluorodecanoic acid	Perfluoroalkyl carboxylic acid	$C_{10}HF_{19}O_2$	335-76-2	5, 8, 9	2, 5, 6, 7, 9	2, 5, 6, 7, 8
PFUnA	Perfluoroundecanoic acid	Perfluoroalkyl carboxylic acid	$C_{11}HF_{21}O_2$	2058-94-8	4, 5, 8	5, 6, 9	5
PFDoA	Perfluorododecanoic acid	Perfluoroalkyl carboxylic acid	$C_{12}HF_{23}O_2$	307-55-1		5, 9	5
PFTrDA	Perfluorotridecanoid acid	Perfluoroalkyl carboxylic acid	$C_{13}HF_{25}O_2$	72629-94-8		5, 9	
PFTeDA	Perfluorotetradecanoic acid	Perfluoroalkyl carboxylic acid	$C_{14}HF_{27}O_2$	376-06-7		5	
PFOSA	Perfluorooctane sulfonamide	Perfluoroalkane sulfonamide	$C_8H_2F_{17}NO_2S$	754-91-6			7
8:2 FTOH	8:2 fluorotelomer alcohol	Fluorotelomer alcohol	$F(CF_2)_8(CH_2)_2OH$	678-39-7	1	7	6, 7, 8
8:2 monoPAP	8:2 polyfluoroalkyl phosphate ester	Polyfluoroalkyl phosphate ester	$F(CF_2)_8(CH_2)_2OP(O)OH_2$	57678-03-2		1	8
8:2 diPAP	8:2/8:2 polyfluoroalkyl phosphate ester	Polyfluoroalkyl phosphate ester	$[F(CF_2)_8(CH_2)_2O]_2P(O)OH$	678-41-1		1	8
8:2 triPAP	8:2/8:2/8:2 polyfluoroalkyl phosphate ester	Polyfluoroalkyl phosphate esters	$[F(CF_2)_8(CH_2)_2O]_3P(O)$	NA		1	8
10:2 diPAP	10:2/10:2 polyfluoroalkyl phosphate ester	Polyfluoroalkyl phosphate ester	$[F(CF_2)_{10}(CH_2)_2O]_2P(O)OH$	1895-26-7		1	8
	**Short Chain PFAS**				**Epidemiological**	**Animal Bioassay**	**In vitro**
PFBS	Perfluorobutane sulfonic acid	Perfluoroalkane sulfonic acid	$C_4HF_9SO_3$	375-73-5	1	2, 8, 10	2, 6, 7, 8

PFHxA	Perfluorohexanoic acid	Perfluoroalkyl carboxylic acid	$C_6HF_{11}O_2$	307-24-4	8	*2*, 8, 10	*2, 8*
PFBA	Perfluorobutanoic acid	Perfluoroalkyl carboxylic acid	$C_4HF_7O_2$	375-22-4		2	8
PFPeA	Perfluoropentanoic aicd	Perfluoroalkyl carboxylic acid	$C_5HF_9O_2$	2706-90-3			5
PFHpA	Perfluoroheptanoic acid	Perfluoroalkyl carboxylic acid	$C_7HF_{13}O_2$	375-85-9			8
GenX (HFPO-DA); PMOH	2,3,3,3-tetrafluoro-2-(1,1,2,2,3,3,3-heptafluoropropoxy) propanoic acid; ammonium salt	Fluorinated ether carboxylate	$C_6HF_{11}O_3$	13252-13-6; 62037-80-3		8	*2*, 8
PMPP/ADONA	3H-perfluoro-3-[(3-methoxypropoxy) propanoic acid, ammonium salt	Fluorinated ether carboxylate	$C_7H_5F_{12}NO_4$	958445-44-8			8
4:2 FTOH	4:2 fluorotelomer alcohol	Fluorotelomer alcohol	$F(CF_2)_4(CH_2)_2OH$	2043-47-2	1	1	8
6:2 FTOH	6:2 fluorotelomer alcohol	Fluorotelomer alcohol	$F(CF_2)_6(CH_2)_2OH$	647-42-7	1	1	8
4:2 diPAP	4:2/4:2 polyfluoroalkyl phosphate ester	Polyfluoroalkyl phosphate ester	$[F(CF_2)_4(CH_2)_2O]_2P(O)OH$	135098-69-0		1	
6:2 diPAP	6:2/6:2 polyfluoroalkyl phosphate ester	Polyfluoroalkyl phosphate ester	$[F(CF_2)_6(CH_2)_2O]_2P(O)OH$	57677-95-9		1	
